# Peptide-based scaffolds for the culture and maintenance of primary human hepatocytes

**DOI:** 10.1038/s41598-021-86016-5

**Published:** 2021-03-24

**Authors:** Douglas MacPherson, Yaron Bram, Jiwoon Park, Robert E. Schwartz

**Affiliations:** 1 Department of Medicine, Weill Cornell Medical College, 413 E 69th St, New York, NY 10021 USA; 2 Department of Physiology, Biophysics, and Systems Biology, Weill Cornell Medical College, 413 E 69th St, New York, NY 10021 USA

**Keywords:** Peptides, Bioinspired materials, Biomimetics, Cell adhesion, Chemical biology, Nanoscience and technology

## Abstract

We report here the use of a nanofibrous hydrogel as a 3D scaffold for the culture and maintenance of functional primary human hepatocytes. The system is based on the cooperative assembly of a fiber-forming peptide component, fluorenylmethyloxycarbonyl-diphenylalanine (Fmoc-FF), and the integrin-binding functional peptide ligand, Fmoc-arginine-glycine-aspartic acid (Fmoc-RGD) into a nanofibrous gel at physiological pH. This Fmoc-FF/RGD hydrogel was formulated to provide a biomimetic microenvironment with some critical features such as mechanical properties and nanofiber morphology, which were optimized to support hepatocyte culture. The material was shown to support maintenance and function of encapsulated primary human hepatocytes as indicated by actin staining, qRT-PCR, and functional cytochrome P450 assays. The designed gel was shown to outperform Matrigel in cytochrome P450 functional assays. The hydrogel may prove useful for liver development and disease models, as well as providing insights into the design of future implantable scaffolds for the regeneration of liver tissue in patients with liver disease.

## Introduction

Cellular function is determined in part by micro-environmental cues such as soluble factors, extracellular matrix, and cell–cell interactions^1,2^. In vitro culture of epithelial cells is often associated with epithelial cell dedifferentiation (i.e. marked by rapid loss of cell-specific morphology, phenotype, and function)^3^. Consequently, there is a need for the development and employment of improved materials which can more closely mimic the in vivo microenvironment and reconstitute the appropriate environmental cues in vitro^1–3^.

Liver disease is an important clinical problem, impacting over 30 million Americans and over 600 million people worldwide, and is the 12th leading cause of death in the United States and 16th worldwide^4^. Due to an aging population and increasing comorbidities including alcohol abuse, viral hepatitis, and obesity, liver morbidity has increased despite improved treatment tools. Given the steady rise in patients with chronic liver disease, the demand for liver transplantation has continued to increase while the supply of organs has remained unchanged, creating a pressing need to address this organ scarcity. An alternative approach to organ transplantation is the transplantation of cells or tissue engineered constructs. The liver is the largest internal organ and the only organ capable of self-regeneration. Owing to the liver’s unique regenerative capacity, a clinically applied scaffold seeded with autologous cells might be able to provide a patient with enough liver tissue for the regenerative mechanisms to take over from the synthetic tissue and produce a functional organ.

We endeavored to use a three-dimensional cell culture environment that more closely mimics the in vivo hepatic microenvironment over traditional two-dimensional tissue-culture plastic models or current 3-D models^5–7^. Previous work in this area has focused on the use of synthetic polymers^8^ or animal-derived materials such as collagen^9,10^ or Matrigel^9^. For example, Matrigel is a cell culture substrate secreted by mouse sarcoma cells and is an undefined mixture composed of a combination of extracellular matrixes and cytokines including collagen and laminin and fibroblast growth factors^11^. More recently, decellularized tissues and organs have also been used as tissue culture scaffolds^12^, but these are animal-derived and are not fully characterized. They may suffer from reproducibility issues and have the possibility of triggering an immune response when introduced to a host^13^. An alternative approach is the use of synthetic polymeric^14^ or supramolecular biomaterials^15^. Peptide-based nanomaterials are especially attractive to mimic a specific natural material and environment, such as an organ or tissue specific extracellular matrix (ECM)^16^. The ECM is a network of proteins and proteoglycans that provide structure and signaling for cells in their natural environment^14^. Using peptide-based nanofibers, researchers have been able to create hydrogels that provide structural support for cells in the laboratory^5,15–22^. With regard to the culture of hepatocytes, peptide-based hydrogels based on the sequence RADA-16^10,22,23^ have been previously utilized.

**Figure 1:**
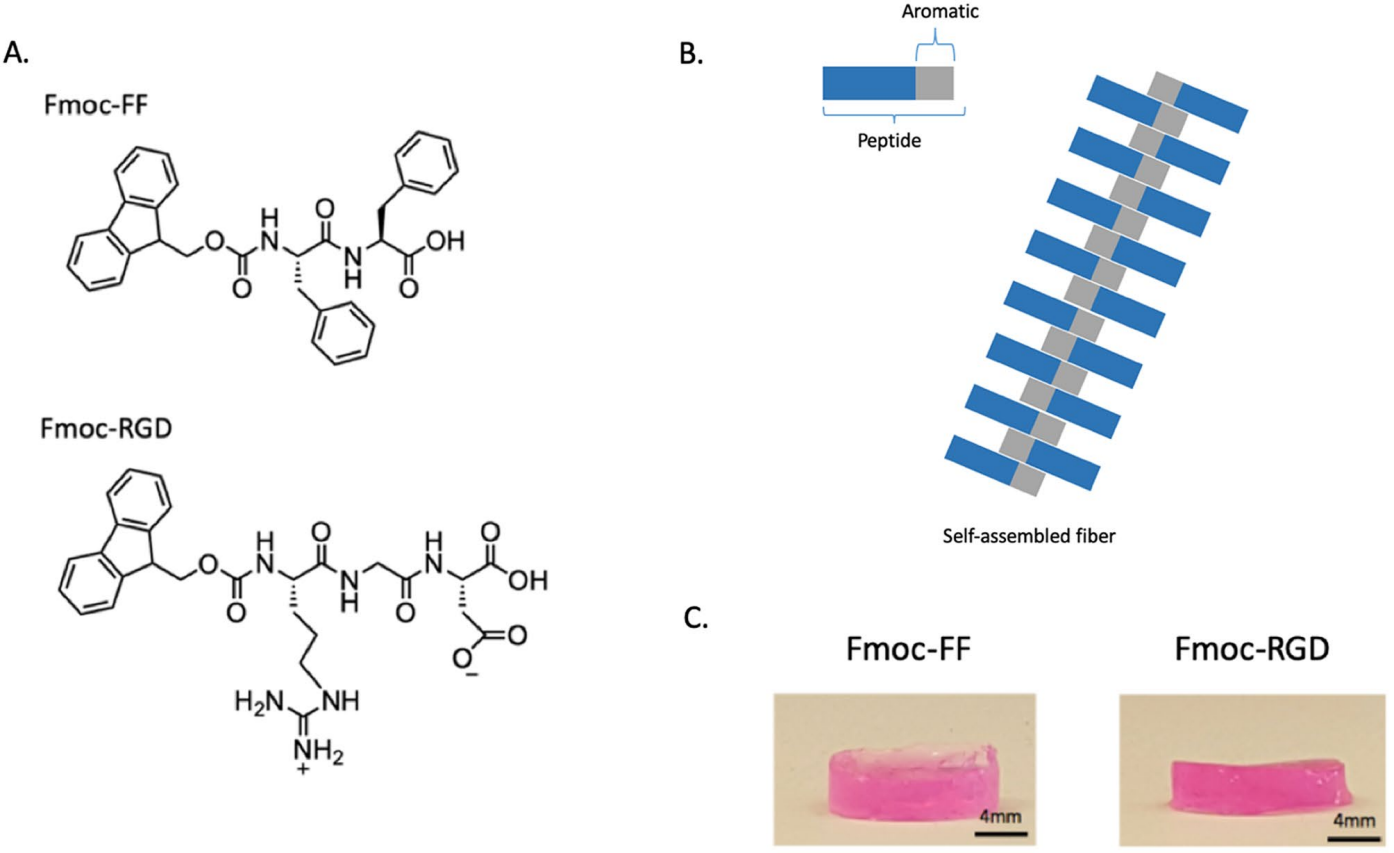
Actin-DAPI staining of primary hepatocyte aggregates cultured in Fmoc-FF/RGD (**A**) Fmoc-FF (**B**) and Matrigel (**C**) hydrogels (scale bars- 50 μm) and assessed for (**D**) cell viability and cytotoxicity. PowerPoint Professional Plus 2013 and Graphpad Version 6.0 was used to make the figure. Structures of the peptides Fmoc-FF and Fmoc-RGD (**A**). Proposed model of peptide fiber assembly adapted from Zhou^27^ (**B**) and 300 μl Fmoc-FF and Fmoc-FF/RGD hydrogel constructs (**C**). Powerpoint Professional Plus 2013 was used to make the figure.

Herein, we report the use of a peptide-based 3D-scaffold for the culture of primary human hepatocytes in which the chemical functionality of the environment was customized for maintenance of hepatocyte function with a focus on matching elastic modulus^24^ and introducing hepatocyte-appropriate adhesion motifs. The materials are based on the co-assembly of a structural, fiber-forming component (fluorenylmethyloxycarbonyl-diphenylalanine, Fmoc-FF) and a functional, adhesion motif (Fmoc-RGD). The fibers are thought to form a morphology which incorporates the RGD ligands at the surface, in a core–shell architecture^25^, (Fig. 1). The system is based on the ability of Fmoc-FF to assemble into a nanofibrous gel at physiological pH^26–29^. While Fmoc-FF gels were shown to support cell culture of chondrocytes^30^, they did not perform well for anchorage dependent cells. Co-assembly with the surfactant Fmoc-serine^25,30^ or the adhesive ligand Fmoc-arginine-glycine-aspartic acid (Fmoc-RGD)^27^ was shown to improve cyto-compatibility.

The signals that the cells receive from their environment are critical for cell survival and functionality. We used the Fmoc-FF architecture combined with the integrin-binding RGD peptide sequence found in the ECM protein fibronectin that signals cells to adhere and proliferate through integrin receptors^31^. Zhou et al. have previously demonstrated the 3D culture of a nonepithelial cell type (primary human dermal fibroblasts) within Fmoc-FF/RGD hydrogel scaffolds^27^, clearly demonstrating the role of RGD in controlling adhesion. Peptide hydrogels decorated with the sequence RGDS have also been employed for tissue engineering scaffolds^32,33^. Peptide hydrogels employing the sequence Fmoc-FRGDF have also been reported^34^. In the current paper, using the Fmoc-FF fiber decorated with the cell adhesion peptide Fmoc-RGD developed by Zhou et al.^27^, we supported hepatocyte survival and function as analyzed through actin staining, enzymatic activity, and maintenance of hepatocyte-specific genes.

## Materials and methods

### Peptide pre-gel solution preparation

Fmoc-FF and Fmoc-FF/RGD peptide powders were supplied by Biogelx Ltd. The powder purities as determined by high performance liquid chromatography (HPLC) were: Fmoc-FF-99.24% (Batch No. FFFMH019); Fmoc-FF/RGD 99.29% and 95.44% (Batch Nos. CUNY0211 and CUNY0236 respectively). 0.04 g of Fmoc-FF/RGD powder in a previously reported^27^ molar ratio of 5:2 Fmoc-FF:Fmoc-RGD was dissolved in 4 mL of deionized water. 0.029 g of Fmoc-FF was dissolved in 3.5 mL deionized H2O and the pH was raised above 10.0 using 0.5 M NaOH to permit solubility, and then lowered to physiological pH using 0.5 M HCl, for a final volume of approximately 4 mL. The Fmoc-FF solution was filtered prior to cell culture. The Fmoc-peptide solutions used for cell culture were sterilized under ultraviolet light for 30 min prior to forming the construct.

### Primary human hepatocytes

Primary human hepatocytes were purchased from vendors permitted to sell products derived from human organs procured in the United States by federally designated Organ Procurement Organizations. Vendors included: Celsis and Bioreclamation Lot numbers NON, NVP, and UBV were used as grouped replicates for all experiments. Normal human hepatocytes were also obtained through the Liver Tissue Cell Distribution System, Pittsburgh, Pennsylvania, which was funded by NIH Contract #HHSN276201200017C. Human hepatocytes were pelleted by centrifugation at 50–100 g for 5–10 min at 4 °C, suspended in hepatocyte culture medium, and assessed for viability using trypan blue exclusion (typically 70–90%). Preparations with less than 90% viability were not used for further studies.

### Cell preparation and cell-gel construct formation

For the 2D micropatterned hepatocyte controls and hepatocyte reference, a micropatterned cocultured platform was used^37^. In brief, off-the-shelf tissue-culture polystyrene or glass-bottom 24-multiwell plates, coated homogenously with rat tail type I collagen (50 μg/mL), were subjected to soft-lithographic techniques to pattern the collagen into microdomains (islands of 500 μm in diameter with 900-μm center-to-center spacing). To create micropatterned cocultures (MPCCs), adult human hepatocytes were seeded on collagen-patterned plates that mediate selective cell adhesion. The cells were washed with medium 2–3 h later (∼4 × 10^4^ adherent hepatocytes in 96 collagen-coated islands in a 24-well plate) and incubated in hepatocyte medium overnight. 3T3-J2 murine embryonic fibroblasts were seeded (9 × 10^4^ cells in each well of a 24-well plate) 24-h later. Hepatocyte culture medium was replaced 24 h after fibroblast seeding and subsequently replaced every day. This platform has been previously shown to maintain robust hepatocyte phenotype and function for up to 4 weeks^37^.

For the 3D constructs, hepatocyte were collected and aggregated as previously described^35^. In brief, to enable 3-dimensional aggregations pre-patterned polydimethylsiloxane (PDMS) wells of 24-well plates were coated with pluronic acid for 2 h at room temperature and the wells subsequently washed three times with DMEM. Primary hepatocytes were combined with J2 murine embryonic fibroblasts at a ratio of 2:1 and aggregated together at a density of 1.2 × 10^5^ total cells/well and 500 μL media/well. All cell culture was performed using culture medium consisting of DMEM with high glucose, 10% FBS and 1% penicillin/streptomycin.

After 96 h of culture on the micropatterned PDMS wells, the cell aggregates (average size 108.2 microns + 9.6 microns^35^) used for gel constructs were introduced to the Fmoc-FF and Fmoc-FF/RGD pre-gel solutions to create hydrogel constructs. This was performed by suspending a centrifuged hepatocyte:J2 cell pellet in a volume of DMEM constituting 10% of the total desired volume for the gel construct. The pre-gel solution was then added and gently pipetted to evenly suspend the cells in the pre-gel solution at the desired concentration of 1 × 10^6^ cells/mL.

300 μL of the above 1 × 10^6^ cells/mL cell-gel solution was added into 1 μm pore ThinCert Transwell cell culture inserts in 12-well plates with 1.4 mL of DMEM added to the outside of the insert in order to induce gelation through electrolyte crosslinking. Therefore 300,000 cells are present in each gel with hepatocytes:J2 at a 2:1 ratio. After 1.5 h of incubation at 37 °C, 300 μL of DMEM was added to the surface of the gel and 1.4 mL was replaced in the well. Cell culture media was changed after 24 h and every 48 h subsequently.

The Matrigel positive control gel constructs were prepared in the same manner as the Fmoc-FF and Fmoc-FF/RGD hydrogels. The Matrigel was thawed and mixed with DMEM in a 1:1 ratio prior to suspending the cells in the gel solution at a density of 1 × 10^6^ cells/mL. 300 μL of the mixture was pipetted into inserts as described above. Therefore 300,000 cells are present in each gel with hepatocytes:J2 at a 2:1 ratio.

### Staining

The gel constructs were fixed in a formaldehyde-sucrose solution for 30 min and permeabilized using a sucrose-NaCl-MgCl2-HEPES buffer for 10 min. Following blocking with bovine serum albumin, 250μL of phalloidin-actin 647 (Invitrogen) stain was added to the gel surface and incubated for one hour. The constructs were washed in Tween solution and mounted using Vectashield mounting medium + DAPI before being compressed between a glass slide and coverslip. Images were acquired with a Zeiss LSM 710 META confocal microscope.

### Cytotoxicity and cell viability assays

Cytotoxicity assays were performed using media supernatants from each gel construct and from 3D aggregates cultured in 2D which were collected at every media change and 15uL from each media change was combined to perform the lactate dehydrogenase LDH-Glo Cytotoxicity Assay (Promega J2380) with analysis performed using a Molecular Devices Spectramax M5. Cell viability assay was performed using the CellTiter-Glo 3D Cell Viability Assay (Promega). In brief the gel constructs and 3D aggregates cultured in 2D were lysed using the CellTiter-Glo 3D reagent and were mixed vigorously for 10 min to induce lysis. The cells were allowed to incubate for an additional 20 min and then luminescent analysis was performed using a Molecular Devices Spectramax M5.

### CYP assays

Cytochrome P450 substrates were mixed into the cell culture media at a concentration of 10 μM and incubated in darkness for two to four hours. Supernatants were collected and processed for fluorescent (BFC, coumarin) or luminescent assays (CYP1A and CYP3A4) as described previously^35–38^ before analysis using a Molecular Devices Spectramax M5. In brief, the substrate medium was high glucose DMEM without phenol red (Gibco) supplemented with 100 units/mL penicillin (Gibco) and 100 μg/mL streptomycin (Gibco). Four substrates divided into two groups were added into medium at proper dilution ratios: group 1 was composed of CYP3A4-IPA for CYP3A4 (dilution: 1:1000, Promega) and 7-benzyloxy-trifluoromethylcoumarin (BFC) (50 μM, Sigma) for multiple CYP450 isoforms; group 2 was composed of 6′deoxyluciferin (Luciferin H) (dilution: 1:50, Promega) for CYP2C9 and Coumarin (50 μM, Sigma) for CYP2A6. The spent medium was removed and the gels were washed with PBS 3 times. The cell aggregates were incubated with group 1 substrate medium for 4 h at 37 °C. After 4 h, the medium was placed in Eppendorf tubes and frozen at − 20 °C for further analysis. The cell aggregates were washed with PBS 3 times and then incubated with group 2 substrate medium for another 4 h at 37 °C. After 4 h, the medium was placed in Eppendorf tubes and frozen at − 20 °C for further analysis. Metabolite conjugates formed from BFC and coumarin were incubated with β -glucuronidase/arylsulfatase (Roche) for 2 h at 37 °C. Samples were diluted 1:1 in quenching solution and formation of metabolites was quantified with a fluorescence microplate reader (Molecular Devices. Metabolite conjugates formed from Luciferin H and CYP3A4-IPA were processed and analyzed per Promega protocol and analyzed using a microplate luminometer (Molecular Devices).

### Alpha-1-Antitrypsin secretion

Alpha-1-Antitrypsin secretion were analyzed by measuring the concentration of Alpha-1-Antitrypsin in culture medium. The medium was collected and replaced with fresh medium every 2 days. The collected medium was centrifuged at 1000 rpm for 5 min. The supernatant was stored at − 20 °C for analysis of Alpha-1-Antitrypsin secretion. Secreted Alpha-1-Antitrypsin in the supernatant was quantified by an enzyme-linked immunosorbent assay (ELISA) kit using sheep anti-human Alpha-1-Antitrypsin antibodies (Bethyl Labs) and horseradish peroxidase detection (3,3′,5,5′-tetramethylbenzidine, Invitrogen).

### RNA extraction and qPCR

RNA was extracted using Trizol and eluted in RNAse-free water. Following RNA extraction, our RNA samples were reverse-transcribed into cDNA using an iScript reverse transcription kit from Bio-Rad. Based on the obtained RNA levels as assessed by a NanoDrop, we diluted the cDNA samples to 15 ng/μL, and used 30 ng total cDNA for qPCR. Primers were used at 100–200 nM, and enough RNase-free water to bring the sample volume up to 15μL per well.

### Biostatistical methods

All samples were repeated in triplicate. Relative gene fold changes for quantitative real time PCR assays were determined through using the 2-ΔΔCt method compared to the β-actin housekeeping gene. Microsoft Excel or GraphPad was used to calculate the P-value test of significance. Data is expressed as the mean + /− standard deviation as described in the text or figure legends.

## Results

### Fmoc-FF/RGD supports hepatocyte survival and functionality

**Figure 2:**
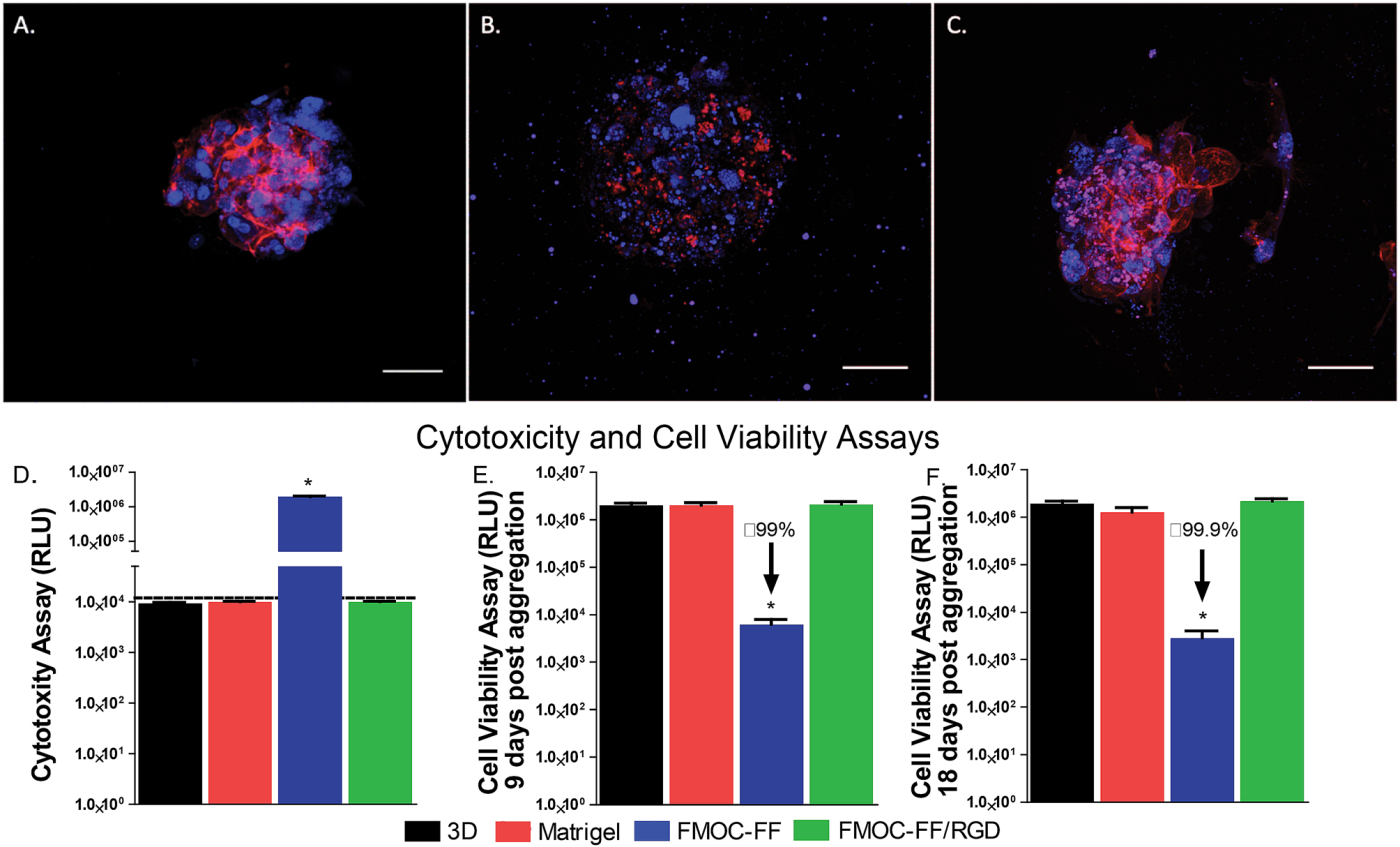
Actin-DAPI staining of primary hepatocyte aggregates cultured in Fmoc-FF/RGD (**A**) Fmoc-FF (**B**) and Matrigel (**C**) hydrogels (scale bars- 50 μm). PowerPoint Professional Plus 2013 and Graphpad Version 6.0 was used to make the figure.

Primary human hepatocytes were utilized to evaluate the 3D culture method in the hydrogel system originally developed by Zhou et al. for the culture of human dermal fibroblasts^27^. The cells were aggregated and seeded into 3D culture in the Fmoc-FF/RGD hydrogels with Fmoc-FF as a negative control and Matrigel acting as a positive control. Actin phalloidin staining was performed five days after initial hydrogel formation, which was designated day zero. Fluorescent imaging revealed comparable morphology and cell survival between Fmoc-FF/RGD and Matrigel, with cells cultured in Fmoc-FF demonstrating significantly less detectable actin signal and evidence of cellular loss (Fig. 2A–C). DAPI staining is also reduced and shows debris in the Fmoc-FF condition suggesting the presence of dying cells (Fig. 2B). Differences in survival between conditions was confirmed using cell cytotoxicity assays which noted significant LDH release in Fmoc-FF cultured aggregates in contrast to Fmoc-FF/RGD, Matrigel, and a 3D hepatocyte aggregate positive control, all of which showed only background levels of LDH release (Fig. 2D). Cell viability assays show maintenance of cell viability for hepatocyte aggregates cultured in Fmoc-FF/RGD, Matrigel and the 3D conditions for 9 and 18 days (5 days and 14 days after incorporation into gels respectively). Consistent with the actin-phalloidin staining, cell viability is low for hepatocyte aggregates cultured in Fmoc-FF hydrogels suggesting that this gel composition cannot support survival of the human hepatocytes (Fig. 2E,F).

Consistent with this finding, alpha-one-antitrypsin (A1AT), one of the top produced proteins by hepatocytes is barely detectable by ELISA for the Fmoc-FF hydrogels (Supplementary Fig. 1). In contrast the FMOC-FF/RGD hydrogels and Matrigel maintained A1AT secretion for 14 days although at levels lower than that of the robust 2D micropatterned hepatocyte controls.

**Figure 3:**
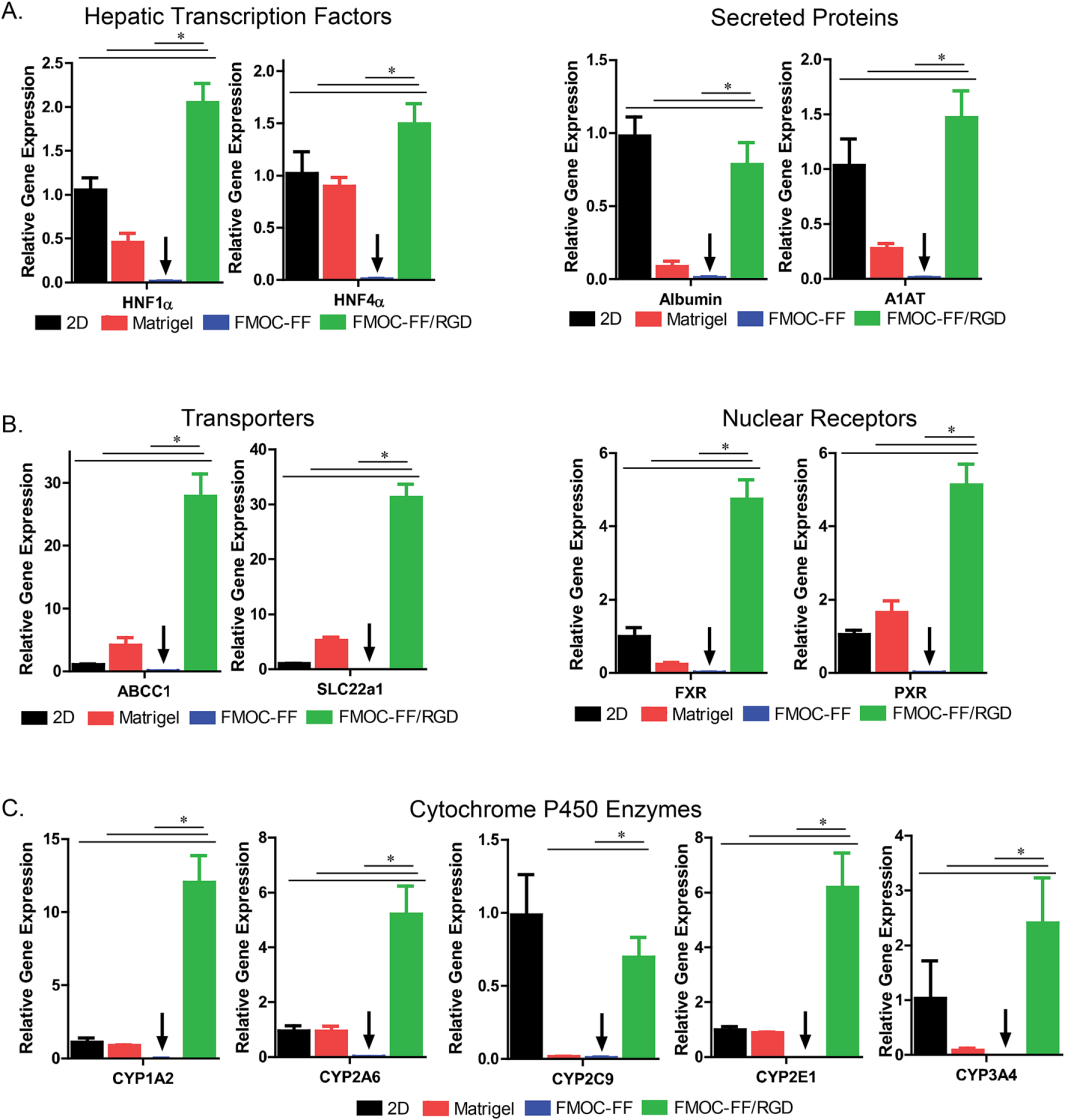
Relative expression of hepatocyte-specific genes in primary hepatocyte cells at day 14 of culture in hydrogel constructs (18 days after initial cell aggregation) Fmoc-FF, Fmoc-FF/RGD, Matrigel control and 2D micropatterned control (and as control reference). Tested genes include hepatic transcription factors HNF1α and HNF4α, secreted proteins albumin and A1AT, transporters ABCC1 and SLC22a1, nuclear receptors FXR and PXR and CYP P450 enzymes 1A2, 2A6, 2C9, 2E1 and 3A4. (*designates *P* < 0.05). N = 3 independent biological replicates. PowerPoint Professional Plus 2013 and Graphpad Version 6.0 was used to make the figure.

qRT-PCR analysis on day 9 and 18 (5 days and 14 days after incorporation into gels respectively) of culture demonstrated significantly higher expression of hepatocyte-specific genes in the primary cells cultured in the Fmoc-FF/RGD gels when compared to the cells cultured in Fmoc-FF (Fig. 3, Supplementary Fig. 2). The categories of genes analyzed include the hepatic transcription factors HNF1α and HNF4α (Fig. 3A, Supplementary Fig. 2A), the secreted proteins albumin and A1AT (Fig. 3A, Supplementary Fig. 2A), the transporters ABCC1 and SLC22a1 (Fig. 3B, Supplementary Fig. 2B), the nuclear receptors FXR and PXR (Fig. 3B, Supplementary Fig. 2B) as well as the CYP P450 enzymes 1A2, 2A6, 2C9, 2E,1 and 3A4 (Fig. 3C, Supplementary Fig. 2C). These genes have been shown to be genes important in defining hepatocyte function and are lost rapidly in traditional culture platforms. Across all genes in all categories, the hepatocytes cultured in Fmoc-FF/RGD hydrogel constructs showed significantly higher gene expression than in Fmoc-FF at 5 days and 14 days after gel incorporation. The Fmoc-FF/RGD condition out-performed Matrigel in all genes with the exception of HNF4α and PXR at 5 days but with all genes at 14 days after gel incorporation. Fmoc-FF/RGD was superior to the 2D micropatterned format which has been previously shown to robustly support hepatocyte gene expression and maintenaince^37^ in all genes except HNF4α, A1AT and CYP3A4 at 5 days and in all genes except Albumin, and CYP2C9 at 14 days (Fig. 3, Supplementary Fig. 2).

**Figure 4:**
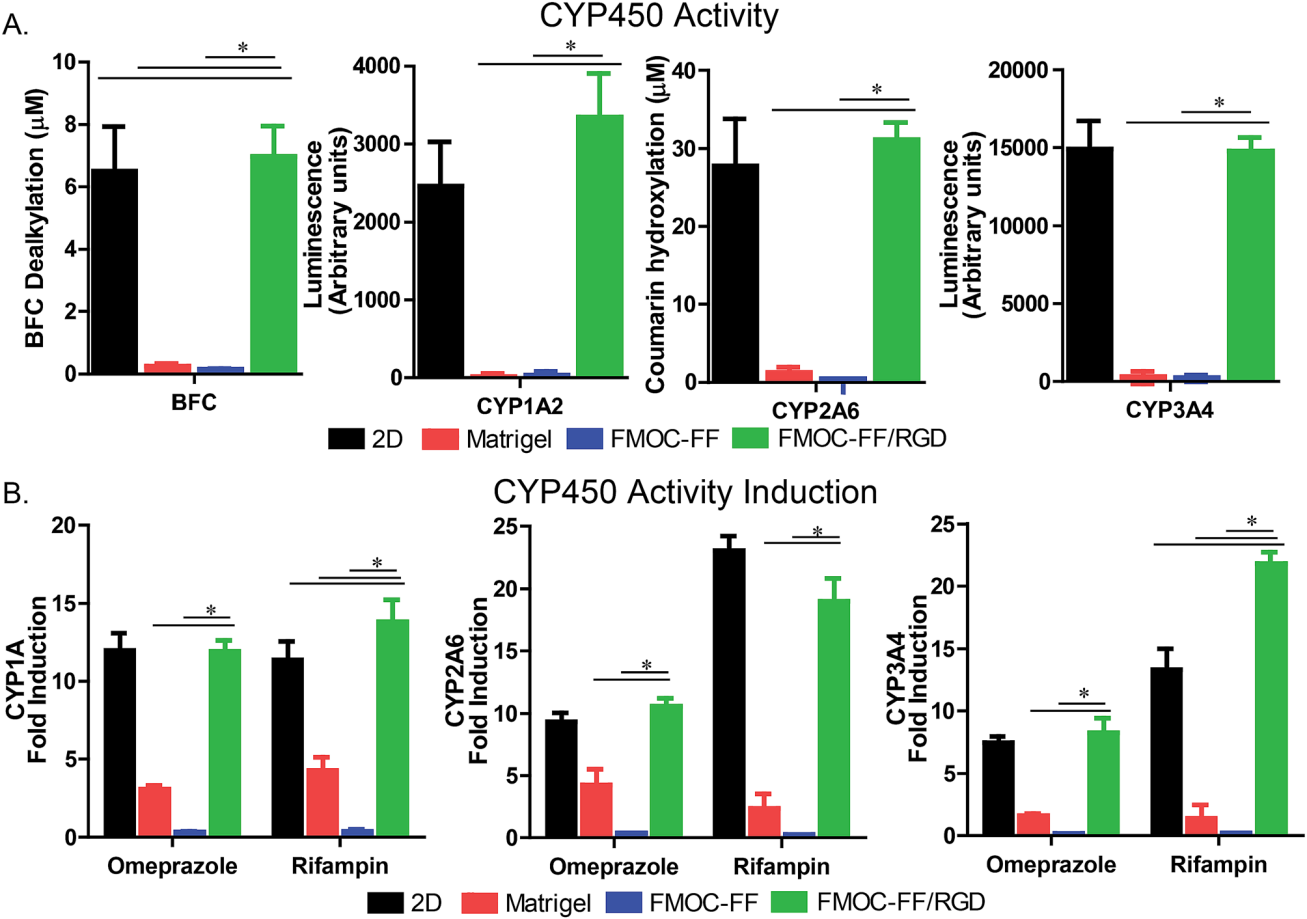
Cytochrome P450 enzyme activity assays for BFC, CYP1A2, CYP2A6 and CYP3A4 for primary human hepatocytes in hydrogel constructs at Day 14 of culture in hydrogel constructs (18 days after initial cell aggregation). (**A**) CYP450 Activity induction assay using Omeprazole and Rifampin for CYP1A, CYP2A6 and CYP3A4 for primary human hepatocytes in hydrogel constructs at day 14 of hydrogel culture. (**B**) (*designates *P* < 0.05). PowerPoint Professional Plus 2013 and Graphpad Version 6.0 was used to make the figure.

Assays for hepatocyte function are critical for determining whether culture platforms maintain not only the phenotype of hepatocytes but the function as well. Functional assays for Cytochrome P450 (CYP) activity for BFC (which assays for multiple cytochrome P450′s), CYP1A2, CYP2A6 and CYP3A4 (which are all cytochrome P450 enzymes that are rapidly lost during hepatocyte dedifferation) on day 5 and day 14 of primary hepatocyte culture showed significantly higher levels of CYP enzyme activity in the Fmoc-FF/RGD hydrogel compared to Fmoc-FF, with the exception of BFC on day 5. Fmoc-FF/RGD-embedded cells had CYP450 activity levels comparable to both Matrigel and the 2D (robust patterned platform previously reported^35,37^) controls at day 5. In contrast by day 14 hepatocytes in Matrigel culture lost most of their CYP450 function as compared to the Fmoc-FF/RGD-embedded cells and the 2D (robust patterned platform previously reported^35,37^) controls. (Fig. 4A, Supplementary Fig. 3). Fmoc-FF/RGD-embedded cells compared well at day 14 the 2D (robust patterned platform previously reported^35,37^) controls.

CYP450 activity induction assay using Omeprazole and Rifampin for CYP1A2, CYP2A6 and CYP3A4 in primary human hepatocytes within hydrogel constructs at day 5 and 14 of culture also reveal significantly higher levels of activity induction for hepatocyte aggregates cultured in the Fmoc-FF/RGD construct when compared to Fmoc-FF (Fig. 4B, Supplementary Fig. 3). The Fmoc-FF/RGD hydrogel also produced higher induction of CYP1A and CYP3A4 activity as compared to Matrigel and comparable levels to the robust 2D system (Fig. 4B, Supplementary Fig. 3).

## Discussion

Self-assembling peptide hydrogels decorated with a functional peptide motif were able to support three-dimensional culture and maintenance of primary human hepatocytes. The researchers who developed this hydrogel thoroughly characterized the material, reporting an elastic modulus between 4 and 9 kiloPascals (kPa)^27^. Our lab confirmed this stiffness range (data not shown). This peptide-based hydrogel system more closely mimics the hepatic microenvironment^39^ as compared to traditional culture methods including tissue-culture plastic (elastic modulus ~ 100,000 kPa) and is comparable to other hydrogel platforms such as Matrigel (average 450 Pa)^40^ or Collagen (0.5–11.5 kPa)^41^.

Cytoskeletal and nuclear staining reveals hepatocytes that are likely dead or dying in the Fmoc-FF negative control, while Fmoc-FF/RGD and Matrigel conditions show cells with defined nuclei surrounded by actin. The cell viability suggested by the actin staining is in agreement with both the cytotoxicity and cell viability assays between conditions. Alpha-one antitrypsin protein production was maintained in Fmoc-FF/RGD constructs as compared to Matrigel and compared favorably to accepted robust micropatterned two-dimensional hepatocyte culture models^35–37^.

qRT-PCR revealed higher level expression and maintenance of hepatocyte-specific genes across several domains of hepatocyte function in Fmoc-FF/RGD when compared to Fmoc-FF for both days 5 and 14. Similarly, gene expression for hepatic nuclear transcription factors, transporters, nuclear receptors, and most cytochrome P450 enzymes were expressed at comparable or higher levels in hepatocytes cultured in Fmoc-FF/RGD constructs compared to Matrigel for both days 5 and 14 and accepted robust micropatterned two-dimensional hepatocyte culture models^35–37^. Relative expression of cytochrome 450 enzymes in the hydrogel construct based on qRT-PCR was significantly higher in hepatocytes cultured in the Fmoc-FF/RGD hydrogel compared to those cultured in the Fmoc-FF construct which was confirmed by cytochrome P450 activity assays for both days 5 and 14. Cytochrome P450 enzyme activity levels at baseline and levels of cytochrome P450 induction of primary human hepatocytes cultured in Fmoc-FF/RGD were similar or superior to those cultured in Matrigel and superior to or comparable to accepted robust two-dimensional hepatocyte culture models^37^. This demonstrates that hepatocytes cultured in the Fmoc-FF/RGD are capable of maintaining CYP450 function without evidence of hepatocyte functional loss.

This 3D system enables the rapid generation of 3D hepatocyte-containing gels with robust cytochrome P450 function in vitro. The co-assembled gels reported here have components which can independently be varied, allowing control over mechanical and biochemical properties to match the needs of specific cell types. The 3D culture method permits the constructs to be scaled up in volume and in number for the purposes of creating a laboratory-based in vitro liver model^42,43^, and in the future, as an implantable scaffold. Future work will involve the differentiation of human pluripotent stem cells into hepatocytes within the Fmoc-FF/RGD construct. Following this, we will pursue the co-culture of the multiple cell types required to create a functional vascularized liver tissue which can then be used as a liver bio-mimetic model platform or in transplantation models. The presented hydrogel system will permit the inclusion of additional functional motifs based on the co-assembly of structural and functional aromatic peptide amphiphiles, to create peptide-based scaffolds for a variety of biomedical applications.

## Supplementary Information

Supplementary Information
