# Supplementary material for: Peptide-based scaffolds for the culture and maintenance of primary human hepatocytes
Source: Sci Rep. 2021 Mar 24;11:6772. doi: 10.1038/s41598-021-86016-5 (PMC7990934; doi:10.1038/s41598-021-86016-5)

Peptide-Based Scaffolds for the Culture and Maintenance of Primary Human Hepatocytes

Douglas MacPherson^a^, Yaron Bram^a^, Jiwoon Park^a^, Robert E. Schwartz^a,b^*

a. Department of Medicine, Weill Cornell Medical College 413 E 69th St, New York, NY 10021,

USA

b. Department of Physiology, Biophysics, and Systems Biology, Weill Cornell Medical College

413 E 69th St, New York, NY 10021, USA

Contact information for authors:

Douglas MacPherson macpherson.douglas@gmail.com 2124133390

Yaron Bram yab2006@med.cornell.edu 6469626197

Jiwoon Park jip2007@med.cornell.edu 6469626197

Robert Schwartz res2025@med.cornell.edu 6469626197

**Supplementary** **Figure 1.** ELISA assays were performed to examine Alpha-1-Antitrypsin secretion by hepatocytes cultured as 2D micropatterned hepatocytes or in aggregates in FMOC-FF, Matrigel, or FMOC-FF/RGD gels. N=3 independent biological replicates. Graphpad Version 6.0 was used to make the figure.


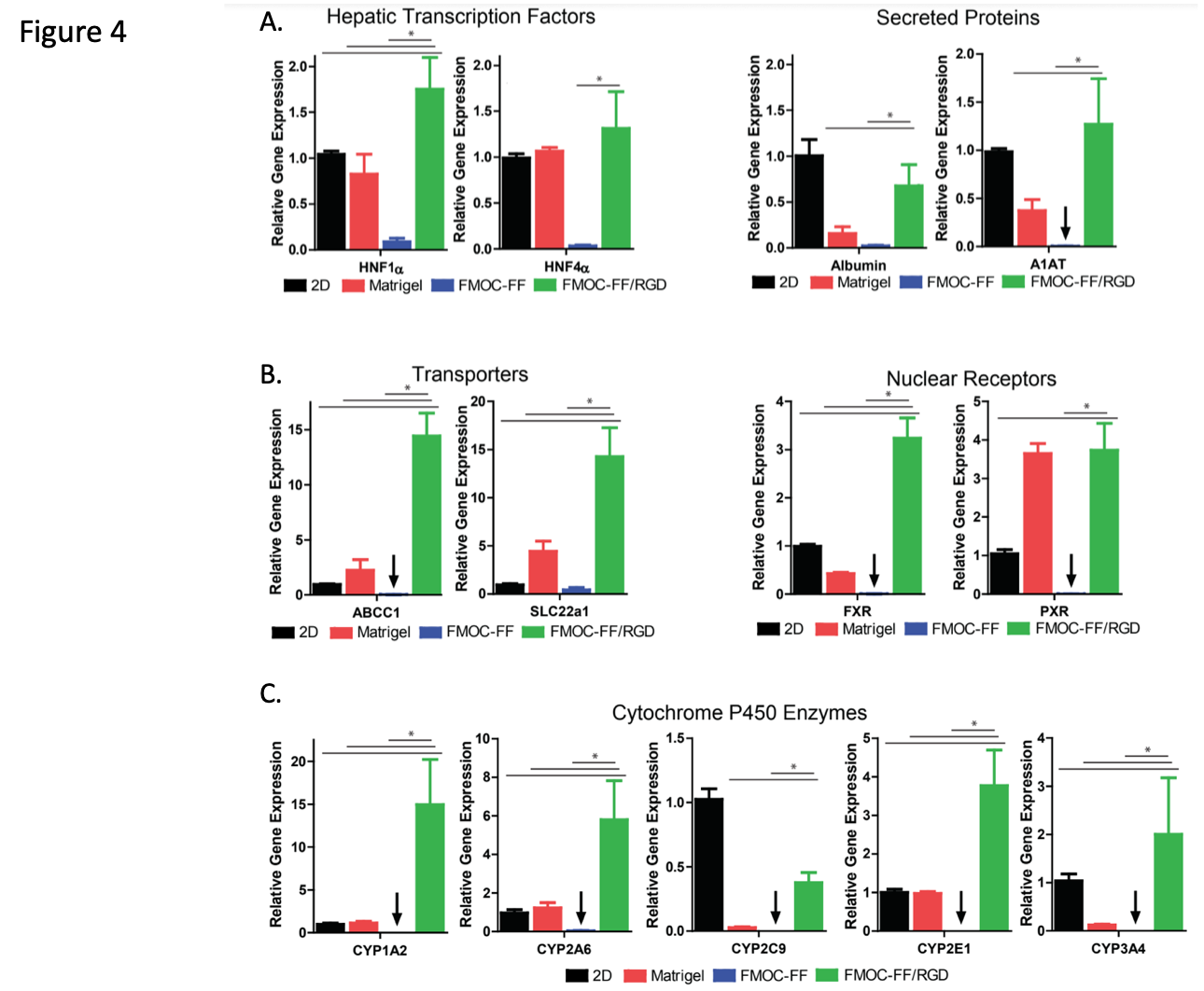


**Supplementary Figure 2.** Relative expression of hepatocyte-specific genes in primary hepatocyte cells at day 5 of culture in hydrogel constructs Fmoc-FF, Fmoc-FF/RGD, Matrigel control and 2D micropatterned control. Tested genes include hepatic transcription factors HNF1α and HNF4α, secreted proteins albumin and A1AT, transporters ABCC1 and SLC22a1, nuclear receptors FXR and PXR and CYP P450 enzymes 1A2, 2A6, 2C9, 2E1 and 3A4. (* designates P < 0.05). N=3 independent biological replicates. Powerpoint Professional Plus 2013 and Graphpad Version 6.0 was used to make the figure.

**Supplementary Figure 3.** Cytochrome P450 enzyme activity assays for BFC, CYP1A2, CYP2A6 and CYP3A4 for primary human hepatocytes in hydrogel constructs at day 4 of culture. (A). CYP450 Activity induction assay using Omeprazole and Rifampin for CYP1A, CYP2A6 and CYP3A4 for primary human hepatocytes in hydrogel constructs at day 4 of culture. (B). (* designates P < 0.05). N=3 independent biological replicates. Powerpoint Professional Plus 2013 and Graphpad Version 6.0 was used to make the figure.


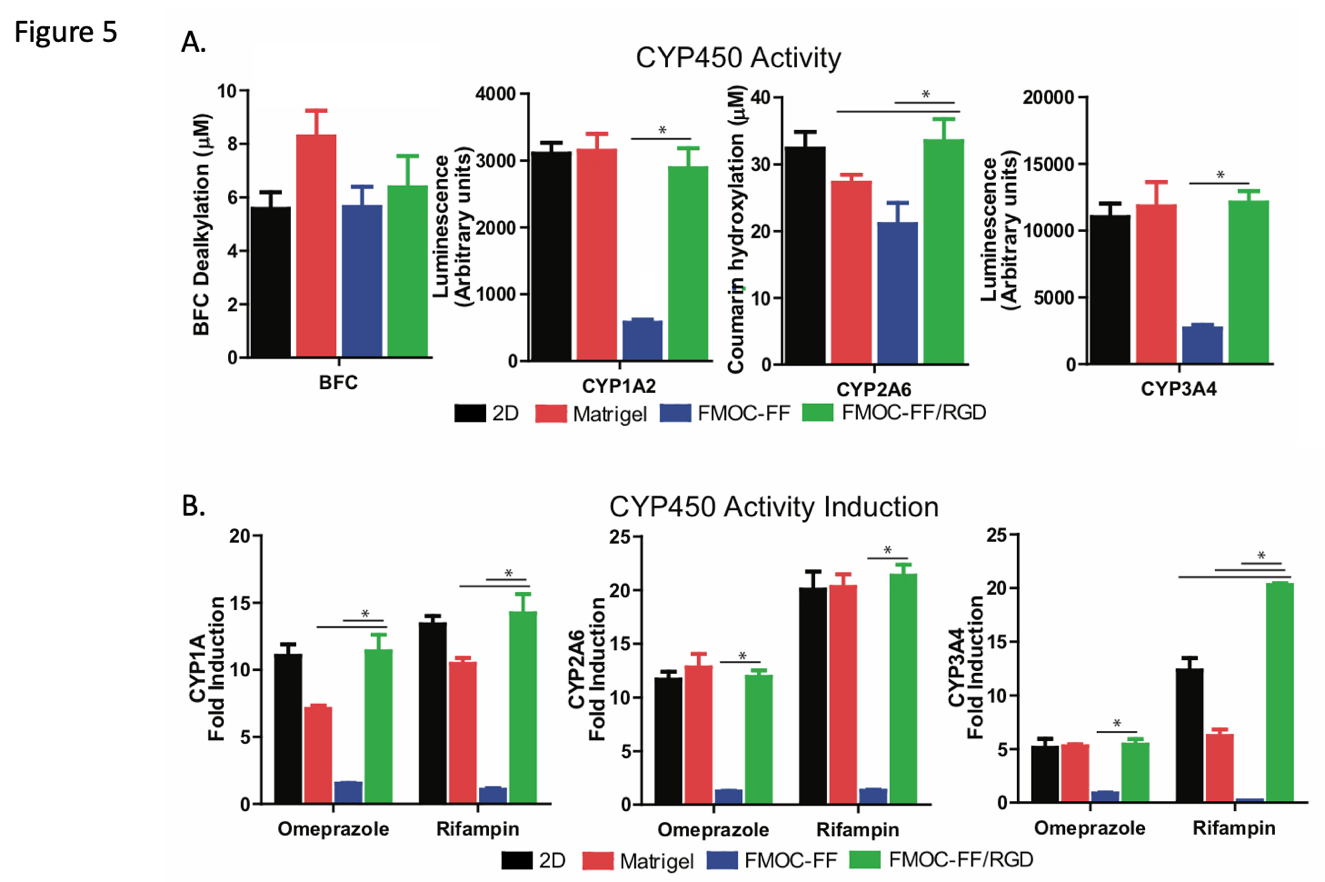

Supplement: Supplementary file 1 — Supplementary Information [file 41598_2021_86016_MOESM1_ESM.docx]
